# Determinants of Poor Quality of Life Among Patients Following Discharge from Acute Psychiatric Units in Alberta

**DOI:** 10.1192/j.eurpsy.2026.11667

**Published:** 2026-07-31

**Authors:** E. Owusu, W. Mao, R. Shalaby, H. E. Elgendy, B. Agyapong, E. Eboreime, M. A. Lawal, N. Nnamdi, P. Chue, A. J. Greenshaw, V. I. Agyapong

**Affiliations:** 1 Psychiatry, University of Alberta, Edmonton; 2 Psychiatry, Dalhousie University, Halifax, Canada

## Abstract

**Introduction:**

Quality of life (QoL) is a multidimensional construct encompassing physical, mental, and social well-being. Understanding the factors that influence QoL is essential for improving post-discharge outcomes among individuals with psychiatric conditions.

**Objectives:**

This study aimed to determine the prevalence and predictors of poor QoL outcomes across the five dimensions of the EQ-5D-5L—mobility, self-care, usual activities, pain/discomfort, and anxiety/depression—among individuals discharged from acute psychiatric care in Alberta.

**Methods:**

Multiple binary logistic regression analyses were conducted to examine associations between sociodemographic characteristics and problems reported in each EQ-5D-5L dimension.

**Results:**

Among 1106 participants, most were Caucasian (61.6%), female (54.8%), aged 25 years or younger (36.4%), and held a high school diploma (51.4%). The prevalence of anxiety/depression was 89.2%. Caucasian participants were twice as likely to report pain/discomfort (OR = 2.14; 95% CI: 1.39–3.27) compared to Black participants. Retired individuals were over three times more likely to experience pain/discomfort (OR = 3.18; 95% CI: 1.45–6.96) than those who were employed. Participants with likely anxiety were nearly twice as likely to report self-care problems (OR = 1.99; 95% CI: 1.41–2.81) compared to those with unlikely anxiety.

**Conclusions:**

The findings reveal a complex interaction between demographic, socioeconomic, and mental health factors influencing QoL among psychiatric patients post-discharge. Tailored, holistic interventions addressing both physical and psychological health are essential to improve recovery and overall well-being in this population.

**Disclosure of Interest:**

None Declared

